# The evolution of parasitism in Nematoda

**DOI:** 10.1017/S0031182014000791

**Published:** 2014-06-25

**Authors:** MARK BLAXTER, GEORGIOS KOUTSOVOULOS

**Affiliations:** Institute of Evolutionary Biology, The University of Edinburgh, Edinburgh EH9 3JT, UK

**Keywords:** Nematoda, nematodes, parasitism, evolution, genome, symbiont, *Wolbachia*, phylogeny, horizontal gene transfer

## Abstract

Nematodes are abundant and diverse, and include many parasitic species. Molecular phylogenetic analyses have shown that parasitism of plants and animals has arisen at least 15 times independently. Extant nematode species also display lifestyles that are proposed to be on the evolutionary trajectory to parasitism. Recent advances have permitted the determination of the genomes and transcriptomes of many nematode species. These new data can be used to further resolve the phylogeny of Nematoda, and identify possible genetic patterns associated with parasitism. Plant-parasitic nematode genomes show evidence of horizontal gene transfer from other members of the rhizosphere, and these genes play important roles in the parasite-host interface. Similar horizontal transfer is not evident in animal parasitic groups. Many nematodes have bacterial symbionts that can be essential for survival. Horizontal transfer from symbionts to the nematode is also common, but its biological importance is unclear. Over 100 nematode species are currently targeted for sequencing, and these data will yield important insights into the biology and evolutionary history of parasitism. It is important that these new technologies are also applied to free-living taxa, so that the pre-parasitic ground state can be inferred, and the novelties associated with parasitism isolated.

## THE DIVERSITY OF THE NEMATODA

Nematoda is an ancient and biologically diverse phylum of moulting animals. They range in size from 0·2 mm to over 6 m, and can be found in most habitats, including within and on host animals and plants (Blaxter and Denver, 2012). In many marine and terrestrial sediments they are the most abundant group in terms of individuals (Platonova and Gal'tsova, 1976), and while only approximately 23 000 species have been described (J. Hallan, unpublished; https://insects.tamu.edu/research/collection/hallan/), the true species-level diversity may be 1 million or more (Lambshead, 1993). Most terrestrial plants and larger animals are associated with at least one species of parasitic nematode, and most of the human population experiences nematode parasitism during their lives (with perhaps one quarter to one third of the global population infected at any time). Estimates of the number of species of parasitic nematode per host suggest that there may be of the order of 25 000 nematode parasites just of vertebrates, most of which remain undescribed (Dobson *et al.* 2008). Nematodes are thus important regulators of plant and animal production. Understanding the evolutionary origins of plant and animal parasitism, and the mechanisms by which parasites locate and invade their hosts, avoid host immunity, and acquire nutrition, are important goals for not only basic, but also for medical and veterinary science. In this paper we discuss the changes in our understanding of the diversity and relationships of nematodes, and of the biology of their parasitic habits, that have been brought about by study of their genes and, increasingly, genomes.

Nematoda are part of Ecdysozoa, a superphylum of animals first defined through analyses of molecular markers (Aguinaldo *et al.* 1997). Support for Ecdysozoa as distinct from other groupings of protostome taxa is less strong from analyses of morphological characters (Nielsen, 2001). Ecdysozoan phyla are characterized by the presence of a cuticle that is periodically moulted during the life cycle, though the specifics of the molecular nature of the cuticle and the orchestration of ecdysis differ between phyla. Other shared features adduced as evidence of relatedness between these phyla include an absence of cilia in adults, and in many members the presence of a triradiate pharynx. The Ecdysozoa in turn comprises two groups, the Panarthropoda (phyla Tardigrada, Onychophora and Arthropoda) and Cycloneuralia (Nematoda, Nematomorpha, Priapulida, Kinorhyncha and Loricifera). Within Cycloneuralia, which may be paraphyletic with respect to Panarthropoda, Nematoda are consistently placed as sisters to Nematomorpha in morphological and molecular analyses (Schmidt-Rhaesa, 1997; Dunn *et al.* 2008).

Nematomorpha are a fascinating group of obligate parasites of terrestrial (Gordioidea) and marine (Nectonematoidea) arthropods. These ‘horsehair worms’ have a parasitoid life cycle, with the larval stages residing within the body cavities of their arthropod hosts, which they kill when they emerge. The adult sexual stages are free-living in pelagic (Nectonematoidea) or sediment (Gordioidea) habitats. Infection of the next host is by ingestion of eggs, often glued to vegetation eaten by the hosts (Hanelt and Janovy, 1999). The generalized life cycle of nematomorphs is very similar to that of mermithid nematodes, which also have marine and terrestrial members, and which also infect their hosts as larvae but have free-living adult stages. The placement of a phylum wherein all members are parasites as sister to all of Nematoda raises the interesting question of whether the ancestor to all nematodes was a parasite (with biology similar to nematomorphs or mermithids), and that the extant free-living groups in Nematoda are reversions from this ancestrally parasitic state. This question has historically been answered in the negative, and molecular data support this conclusion, because free-living nematodes arise basally to Mermithida in Nematoda. The similarity in lifestyle is thus most likely to be homoplasious (i.e. has arisen independently by convergent evolution).

**Fig. 1.**
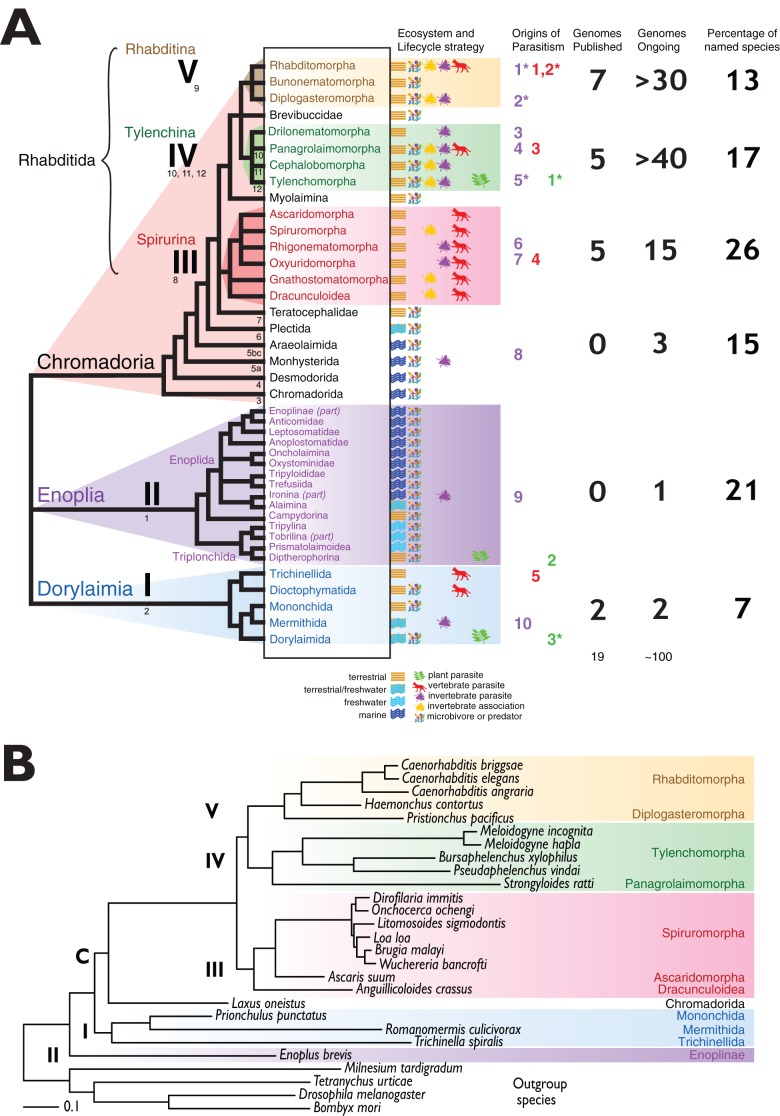
The phylogenetic structure of the Nematoda and the origins of parasitism (A) A cartoon of the phylogenetic structure of the Nematoda, based on nuclear small subunit ribosomal RNA analyses and interpretation of taxon relationships derived from morphology (De Ley and Blaxter, 2004; Blaxter and Denver, 2012). Taxon systematic names are given for the major nodes in the phylogeny. Clades I, II, C, III, IV and V were first defined in Blaxter *et al.* (1998). Helder and colleagues revised the numbering of clades (Holterman *et al.* 2006; van Megen *et al.* 2009), and their schema is given in smaller Arabic numerals beneath the relevant branches. For each ordinal/subordinal group named, the ecosystem and trophic habits are indicated by small icons. For the major clades, the numbers of published genomes, genomes in progress and the proportion of named species (Hallan, 2007) are given. (B) The utility of large scale nematode genome data for phylogenetic analyses. A phylogeny of Nematoda derived from 181 protein coding genes from 23 nematode species, and four ecdysozoan taxa as outgroup. The alignment was subjected to analysis with PhyloBayes (Lartillot *et al.* 2009), and all nodes had posterior probability of 1·00. The major clades in Rhabditida are resolved, and Enoplia is recovered at the base of Nematoda. The figure is adapted from Blaxter *et al.* (2014).

The molecular systematics of the Nematoda have been explored for nearly 20 years (Blaxter *et al.* 1998; Kampfer *et al.* 1998), and comprehensive analyses of the breadth of diversity of the phylum now converge on a stable phylogeny (Meldal *et al.* 2007; Holterman *et al.* 2009; van Megen *et al.* 2009; Bik *et al.* 2010). These analyses have largely used the nuclear small subunit ribosomal RNA gene (nSSU), as it combines features of conservation and change that are informative over deep timescales. New genomic data are being brought to bear on the phylogenetics of Nematoda, and revisions of the tree may still be necessary (see below). While most traditional analyses suggested a bipartite division of the phylum, into ‘Adenophorea’ (largely marine, but also including terrestrial plant and animal parasites) and ‘Secernentea’ (largely terrestrial, and including many animal and plant parasites), the molecular analyses show three major divisions (Fig. 1A). The ‘Adenophorea’ are split between these three divisions, and ‘Secernentea’ are a subgroup of one. The new phylogeny was sytematized by De Ley and Blaxter (2002, 2004).

Nematoda comprises the subclasses Enoplia, Dorylaimia and Chromadoria (De Ley and Blaxter, 2002, 2004). In nSSU analyses the branching order of these three groups is unresolved, though there are hints that Enoplia may be the earliest-branching of the three (van Megen *et al.* 2009; Blaxter *et al.* 2014). The inability of nSSU to robustly distinguish the branching order and thus the root of the phylum is due to lack of strong signal, exacerbated by the phylogenetic distance to the nearest outgroup taxa (other Ecdysozoa, which likely last shared a common ancestor well before the Cambrian, over 540 My ago).

It is generally argued that Nematoda has a marine origin (see Fig. 1A). The Enoplia are largely marine, and mostly free-living. They are the commonest nematodes in marine sediments, and dominate deep-sea ecosystems where they feed on diatoms and marine algae. Members of Enoplia are also found in brackish and fresh water, and on land, including plant parasites. The Dorylaimia are freshwater or terrestrial nematodes and include major groups of plant and animal parasites. The Chromadoria includes a large number of marine groups, and a major terrestrial radiation that includes plant and animal parasites. In Chromadoria the terrestrial taxa appear to have arisen from marine ancestors, but the situation in the other subclasses is less clear. Dorylaimia has few truly marine taxa, and in Enoplia molecular phylogenies place the terrestrial/freshwater Triplonchida as sister to the remaining (marine) Enoplida.

## THE MULTIPLE ORIGINS OF PARASITISM WITHIN THE NEMATODA

Nematodes exhibit a wide range of relationships with other species. Parasitism is a common way of life, and a large proportion of nematode species may be parasites. Poulin has usefully classified the different kinds of parasitic relationships between species into a spectrum of life-habit modes (Poulin, 2011; Poulin and Randhawa, 2013). Some relationships are phoretic: the nematodes use another species to aid dispersal to new sites (Bovien, 1937). Phoretic associations can be very specific, as in *Rhabditis stammeri*, associates of burying beetles (*Nicrophorus* spp.), which have a complex response to the beetle life cycle that assures their presence in emerging adults by entering and diapausing in the hind guts of mature larvae before they pupate (Richter, 1993). Other phoretic relationships are less specific, and dispersal stages can be found attached to many different transport hosts. In most cases, the dispersal stage is a third stage juvenile (J3, or L3 for larva). The costs to the phoretic host are hard to measure, but may be significant in heavily colonized hosts, or where the associate extracts some nutrition from its carrier. Many plant parasitic nematodes, particularly the migratory endoparasites, could be classified as microherbivores (Poulin and Randhawa, 2013), as their ecology is similar to that of an ungulate browsing on bushy plants. However these nematodes do induce specific cell responses in host plants, and thus the relationship is more than just browsing. Some intestinal parasites, such as the Rhigonematida of millipedes (Hunt, 1996), feed on gut contents or other nematode parasites rather than on host tissue, and might even be classed as commensals.

Independent origins of the parasitic habit can be validated by molecular phylogenetic placement of parasitic taxa and their free-living relatives (Blaxter *et al.* 1998; Dorris *et al.* 1999). With the current available molecular data we can define three origins of plant parasitism, 10 of parasitism of a wide range of non-vertebrates and five of parasitism of vertebrate hosts across the three subclasses (Fig. 1 and Table 1). Many additional events of acquisition of parasitic lifestyles could be proposed. For example, Sudhaus has suggested at least 20 independent events of acquisition of parasitism of insects in Nematoda (Sudhaus, 2008). Enoplia has the fewest parasitic species, while Chromadoria (and Rhabditida within Chromadoria) has the most. All parasitic groups appear to have a terrestrial or limnic origin. The sole enoplian animal parasite, *Ironus macrocephalum* (Ironidae), was described from the earthworm *Pheretima wendessiana* in New Guinea (Pierantoni, 1916). Other Ironidae are freshwater species.

**Table 1.** Origins of parasitism in the Nematoda^a^

Origin number^b^	Nematode group	Host group(s)	Parasitic types sensu Poulin^c^	Comments
Non-vertebrate hosts				
1 (multiple events)	*Heterorhabditis, Phasmarhabditis, Rhabditis* (and others)	Hexapoda, Mollusca, Clitellata	Parasitoid, Castrator, Directly transmitted parasite	Rhabditomorpha contains many taxa with phoretic relationships with arthropods and molluscs
2 (multiple events)	Diplogasteromorpha (e.g. *Parasitodiplogaster, Medhinema, Cephalobium*)		Directly transmitted parasite	Diplogasteromorpha contains many species with phoretic or necromenic associations with arthropods
3	Drilonematomorpha	Clitellata	Directly transmitted parasite	
4	Steinernematidae, Allantonematidae	Hexapoda	Directly transmitted parasite	Some species show ‘alternation of generations’ where the parasite can reproduce both within and outside the host
5 (multiple events)	Hexatylina, Aphelenchoidea, Sphaerularoidea (and others)	Hexapods	Castrator, Directly transmitted parasite	*Daubaylia*, a parasite of snails, may be a member of Tylenchomorpha
6	Rhigonematiomorpha	Myriapoda	Directly transmitted parasite	Rhigonematomorpha is nested within the otherwise vertebrate parasitic Spirurina; the group contains no vertebrate parasites
7	Oxyuridomorpha	Arthropoda	Directly transmitted parasite	Oxyuroidomorpha is nested within the otherwise vertebrate parasitic Spirurina; the group also contains vertebrate parasites
8	Monhysterina (*Gammarinema*)	Crustacea	?Directly transmitted parasite	May be classed as ‘commensals’ rather than parasites
9	Ironina	Annelida	?Directly transmitted parasite	
10	Mermithida	Arthropoda	Parasitoid	
Vertebrate hosts				
1	Strongyloidea	Vertebrates	Directly transmitted parasite, Trophically transmitted parasite, Vector transmitted parasite	Some species have non-vertebrate paratenic or vector hosts; *Heterorhabditis* is a sister group to the vertebrate parasites
2 (multiple events)	Rhabdiasidae, *Pelodera strongyloides*	Anuran	Directly transmitted parasite	There may be additional independent he precise placement of Rhabdiasidae awaits resolution
3	Strongyloididae	Mammals	Directly transmitted parasite	‘Alternation of generations’ where the parasite can reproduce both within and outside the host
4	Spiruromorpha	Vertebrates	Directly transmitted parasite, vector transmitted parasite, trophically transmitted parasite	Includes the non-vertebrate parasitic oxyurids and rhigonematids
5	Trichinellida plus Dioctophymatida	Mammals	Directly transmitted parasite, trophically transmitted parasite	
Plant hosts				
1 (?multiple events)	Tylenchomorpha	Viridiplantae (and macroalgae)	Directly transmitted parasite, Micro-predator	Plant parasitic groups are associated with fungal-feeding groups, and there may be an association; plant parasitism may have arisen multiple times
2	Diptherophorina (Trichodoridae)	Viridiplantae	Directly transmitted parasite, Micro-predator	
3 (?multiple events)	Dorylaimida (*Xiphinema, Longidorus* and others)	Viridiplantae	Directly transmitted parasite, Micro-predator	Plant parasitism may have arisen multiple times

a There are many isolated additional descriptions of nematode associations with other taxa.

b The numbering of events follows Fig. 1.

c See Poulin (2011); Poulin and Randhawa (2013) for details.

There are some striking phylogenetic associations between non-vertebrate and vertebrate parasites. The Strongylomorpha (in Rhabditina), a major group of gut and airway parasites of vertebrates, are sisters to the Heterorhabditidae. *Heterorhabditis* species are entomopathogens that invade the haemocoel of insect larvae, and release a symbiotic bacterium that kills the host. Similarly, the mammal-parasitic Strongyloidoidae (Tylenchina; Panagrolaimomorpha) are related to the entomopathogenic Steinernematidae. *Steinernema* species also invade the haemocoel, and use a bacterial symbiont (a different group of species) to kill their hosts. The entomopathogens show convergence in life style, and are phylogenetically associated with a transition to vertebrate parasitism. However, there is no evidence that the vertebrate parasites utilize symbiotic bacteria during their life cycle. One model for the origin of the vertebrate-parasitic Strongylomorphs and Strongyloidoids is that they represent host capture by an ancestral terrestrial, entomopathogenic or entomoparasitic species, and subsequent radiation in the new host groups. Not all associations with arthropods necessarily lead to full parasitism, as there are several nematode groups (notably the Diplogasteromorpha in Rhabditina) where many species are phoretic or necromenic associates of arthropods but very few parasites have been found.

The Spirurina (Chromadoria; Rhabditida) are all parasites, mostly in vertebrates. Many Spirurina utilize vector hosts to facilitate transit from one definitive host to another, but there are also groups (Ascaridomorpha, Oxyruidomorpha) that do not use intermediate hosts. Some groups utilize multiple intermediate hosts, with Gnathostomatomorpha passaging through both a first, crustacean paratenic host and a second, fish host before establishing in carnivorous mammals. Surprisingly, in the new Spirurina phylogeny, groups with a simple, direct life cycle appear to be derived from a radiation of groups that have vector hosts. Gnathostomatomorpha arise basally, and Ascaridomorpha and Oxyuridomorpha have their origins as sisters to other vector-borne groups such as Spiruromorpha (Nadler *et al.* 2007; Laetsch *et al.* 2012). Loss and gain of vector hosts within groups are common, and radical shift of vector species while continuing to use similar definitive hosts is a common feature of related parasites. For example, the Onchocercinae, parasitizing rodents, ungulates and primates, utilize mosquitoes, tabanid flies, black flies and mites as vectors. Two groups of arthropod parasites are nested within the otherwise vertebrate parasitic Spirurina (Rhigonematomorpha are gut parasites of large millipedes, and many Oxyuridomorpha are parasites of arthropods), and thus there must have been at least two independent transitions from vertebrate to arthropod parasitism (possibly by neotenic development in vector species) in these groups. Interestingly, *Philometra obturans*, a dracunculoid parasite of pike, has been observed to grow to sexual maturity in copepod intermediate hosts when the fish definitive host is absent (Moravec and de Buron, 2013).

## COMMON THEMES

There are few common themes in the parasitic lifestyles of nematodes. One is that the stage at which parasites transition from a free-living portion of the life cycle to the parasitic portion (or vice versa) is usually the J3/L3 stage, the same stage usually involved in phoretic associations (Sudhaus, 2010). Most rhabditid parasites first invade their hosts as J3, and mermithids exit their hosts as J3. The diapausing J3 stage of the free-living nematode *Caenorhabditis elegans* (not a parasite, but a species that has phoretic associations with terrestrial isopods) is particularly resistant to environmental insult, and is used as a model for the infective J3 of parasitic nematodes (particularly in Rhabditomorpha). It has been suggested that adaptation to rich food sources low in available oxygen such as rotting vegetation might predispose some groups to the acquisition of parasitic habits as the guts of other animals might offer a similar set of environmental challenges.

Echoes of the ‘great chain of being’, a world view common since pre-Darwinian times but still expressed today, can be heard in many discussions of parasite evolution. Parasites are suggested to be likely to show regression, losing morphological and metabolic complexity as they come to rely on hosts for more and more of the details of life as a metazoan. However, while a gut parasite might rely on the host for metabolic provisioning (thus implying a scope for simplification), it may also be subject to new stresses and demands in terms of countering host immunity (implying evolution of novelty). Similarly, parasites that utilize multiple hosts, or have major transitions in lifestyle, might be expected to gain or at least retain metabolic complexity, as they require toolkits to thrive in several distinct environments. Morphological novelties are common in parasitic nematodes: buccal teeth in hookworms (Chromadoria; Strongylomorpha); tagmatization in *Trichuris* (Dorylaimia; Trichinellidae); ornate spines, flanges and other cuticular decorations observed in arthropod-parasitic Oxyuridomorpha and Rhigonematomorpha (Chromadoria; Spirurina).

Even parasites with apparently simple life cycles can have very complex biology. Most Ascaridids infect new hosts through ingestion of embryonated eggs from the environment, and are often used as examples of simple, direct life cycles. *Ascaris suum*, which enters the host intestine as larvae arrested within a protective eggshell, but then invades the gut wall, enters the bloodstream, and reinfects the gut by exiting from the capillaries in the lungs, climbing the tracheal tree and returning to the gut by being swallowed, has a very complex in-host life cycle. This migration seems against reason, as it is costly to the parasite, and has been argued to be retention of an ancient trait of migration derived from a vector-borne ancestor. Retention of a costly phenotype in contemporary species requires explanation. The answer, derived by analysis of species pairs that differ in whether they migrate or not, is simply that migration is strongly associated with adult body size and with adult fecundity: migration is an adaptive trait maintained by selection (Skorping *et al.* 1991; Read and Skorping, 1995*a*, *b*). It may be that the ability to migrate was inherited from an ancestral species, but its presence in living taxa is because it is currently adaptive.

## GENOMICS AND PARASITISM

The first metazoan genome to be sequenced was that of a nematode: *C. elegans* (Chromadoria; Rhabditomorpha) (The *C. elegans* Genome Sequencing Consortium 1998) and nematode parasitologists were early adopters of the genomics toolkit (Blaxter *et al.* 1996, 1997). A major effort over the past 20 years has delivered a large body of data on the transcribed genes of nematodes, initially through Sanger dideoxy sequencing expressed sequence tag sampling approaches (Parkinson *et al.* 2004) but latterly using the new sequencing technologies to deliver whole transcriptomes (Blaxter *et al.* 2012). The roster of nematode species with significant transcriptome data publicly available now exceeds 100. These data have been critical in revealing genes associated with parasitic traits, and in identifying new drug targets and vaccine candidates (Elsworth *et al.* 2011).

The first parasitic nematode genome to be sequenced was that of *Brugia malayi* (Chromadoria; Spiruromorpha) (Ghedin *et al.* 2007). To date 19 nematode genome sequences have been published (see Fig. 1A and Table 2), and the majority of these genomes derive from parasites. As might be expected there is a bias in the species chosen for sequencing, and vertebrate and especially human parasites are over-represented. New sequencing technologies allow a new draft genome to be generated with very little resource, and more and more groups are now exploring the genomes of their target taxa. A loose alliance of nematode genome-interested biologists has been founded, and is nucleating the sequencing of as many genomes across the diversity of the phylum as is possible. The 959 Nematode Genomes initiative (Kumar *et al.* 2012*a*, *b*) mimics other community genome programmes such as the insect 5k project (i5K Consortium, 2013) (excepting that the headline goal is derived from the number of somatic cells in the adult female hermaphrodite *C. elegans*). It allows contributors to post information on the genomes they are pursuing and update the community as to their successes, and is a clearing-house for queries and collaborations (see http://www.nematodegenomes.org). By showing that genome sequencing, assembly, annotation and interpretation are within the reach of any research community, the initiative now has recorded well over 100 species for which genome data have been or are being generated.

**Table 2.** Nematode genome sequences

Species	Systematic position	Genome size (Mb)^a^	Contiguity of assembly N50^b^ (kb)	Number of scaffolds	Number of nuclear protein-coding genes predicted	Web link for access (publication)
Clade I: Dorylaimia						
*Romanomermis culicivorax*	Mermithida	322·77	17 632	62 537	10 206	http://romanomermis.nematod.es (Schiffer *et al.* 2013)
*Trichinella spiralis*	Trichinellida	63·51	6 373 445	6819	16 380	www.wormbase.org/species/t_spiralis (Mitreva *et al.* 2011)
Clade III: Spirurina						
*Acanthocheilonema viteae*	Spiruromorpha	77·35	25 808	6796	10 397	http://acanthocheilonema.nematod.es http://badger.bio.ed.ac.uk/filarial/
*Ascaris suum*	Ascaridomorpha	334^c^	290 558	260	15 446	http://ascaris.nematod.es (Wang *et al.* 2012)
*Brugia malayi*	Spiruromorpha	94·14	191 089	9827	17 846	www.wormbase.org/species/b_malayi (Ghedin *et al.* 2007)
*Dirofilaria immitis* (and *Wolbachia wDi*)	Spiruromorpha	88·30 (0·92)	22 560 (919 954)	71 281 (2)	16 061 (871)	http://dirofilaria.nematod.es http://badger.bio.ed.ac.uk/filarial/ (Godel *et al.* 2012)
*Litomosoides sigmodonti (*and *Wolbachia wLs)*	Spiruromorpha	64·81 (1·05)	45 863 (605 213)	3165 (10)	10 246 (1042)	http://litomosoides.nematod.es http://badger.bio.ed.ac.uk/filarial/ (Comandatore *et al.* 2013)
*Loa loa*	Spiruromorpha	91·37	174 388	5770	15 444	www.broadinstitute.org/annotation/genome/filarial_worms (Desjardins *et al.* 2013)
*Onchocerca ochengi (*and *Wolbachia wOo)*	Spiruromorpha	95·51 (0·96)	12 317 (957 990)	24 057 (1)	13 990 (664)	http://onchocerca.nematod.es http://badger.bio.ed.ac.uk/filarial/ (Darby *et al.* 2012)
*Wuchereria bancrofti*	Spiruromorpha	81·51	5161	25 884	19 327	www.broadinstitute.org/annotation/genome/filarial_worms (Desjardins *et al.* 2013)
Clade IV: Tylenchina						
*Panagrellus redivivus*	Panagrolaimomorpha	65·06	267 941	867	24 249	www.wormbase.org/species/p_redivivus (Srinivasan *et al.* 2013)
*Bursaphelenchus xylophilus*	Tylenchomorpha	74·56	949 830	5526	18 074	www.wormbase.org/species/b_xylophilus (Kikuchi *et al.* 2011)
*Meloidogyne hapla*	Tylenchomorpha	53·02	37 608	3452	14 420	www.wormbase.org/species/m_hapla (Opperman *et al.* 2008)
*Meloidogyne incognita*	Tylenchomorpha	82·09	12 786	9533	19 212	www.wormbase.org/species/m_incognita (Abad *et al.* 2008)
*Meloidogyne floridensis*	Tylenchomorpha	96·67	3698	58 696	11 975	http://meloidogyne.nematod.es (Lunt *et al.* 2014)
Clade V: Rhabdina						
*Caenorhabditis angaria*	Rhabditomorpha	99·01	87 708	11 453	27 967	www.wormbase.org/species/c_angaria (Mortazavi *et al.* 2010)
*Caenorhabditis briggsae*	Rhabditomorpha	108·42	17 485 439	12	21 850	www.wormbase.org/species/c_briggsae (Stein *et al.* 2003)
*Caenorhabditis elegans*	Rhabditomorpha	100·29	17 493 829	7	20 520	www.wormbase.org/species/c_elegans (The *C. elegans* Genome Sequencing Consortium 1998)
*Caenorhabditis sp 1*	Rhabditomorpha	109·33	24 542	14 350	^d^	http://caenorhabditis.nematod.es
*Caenorhabditis sp 5*	Rhabditomorpha	131·80	25 228	15 261	46 280^d^	http://caenorhabditis.nematod.es
*Caenorhabditis sp 16*	Rhabditomorpha	86·00	57 356	8419	^d^	http://caenorhabditis.nematod.es
*Pristionchus pacificus*	Diplogasteromorpha	170·36	1 290 309	10 227	24 217	www.wormbase.org/species/p_pacificus (Dieterich *et al.* 2008)
*Dictyocaulus viviparus*	Rhabditomorpha	169·39	22 560	17 715	14 306	http://dictyocaulus.nematod.es (Koutsovoulos *et al.* 2014)
*Haemonchus contortus*	Rhabditomorpha	368·83	83 501	19 726	24 775	www.wormbase.org/species/h_contortus (Laing *et al.* 2013)
*Heterorhabditis bacteriophora*	Rhabditomorpha	77·01	312 328	1259	20 964	www.wormbase.org/species/h_bacteriophora (Bai *et al.* 2013)

a Genome size is estimated from the span of genome assembly.

b The N50 is the weighted median scaffold length (the scaffold length at which 50% of the assembled genome is in scaffold of that length or greater).

c The *A. suum* genome undergoes chromatin diminution such that the somatic genome is ~40 Mb smaller than the germline genome (Wang *et al.* 2012).

d The gene predictions for *Caenorhabditis* sp. 5 are preliminary. The gene sets for the *Caenorhabditis* species are being re-predicted as part of a co-analysis across 10 *Caenorhabditis* genomes (E. Schwarz, M. Blaxter, unpublished).

We have been working to deliver genomic data for a number of species of key interest. These include free-living species (for example representatives of the under-sampled Enoplia, and additional *Caenorhabditis* species that will illuminate the biology of the model *C. elegans*) as well as parasites (Table 2). Using short-read next-generation sequencing technologies, and freely available assembly and annotation toolkits, we have been able to deliver important new genomes to the research communities. While generation of genomes of the quality and completeness of *C. elegans* would still require very significant effort, producing an assembly that is verifiably near-complete (albeit fragmented), and a gene set that is useful for a wide range of subsequent analyses, is relatively routine. A major remaining issue is that of heterozygosity in wild-sourced specimens. The *C. elegans* genome was derived from an essentially homozygous matrilineal clone, and the *B. malayi* genome from a highly inbred stock. Wild-caught, or recently colonized, nematode species will carry high levels of heterozygosity, and several animal and plant-parasitic nematode genome projects have struggled with the issues that extreme levels of heterozygosity (even in parasite lines maintained for a long time in laboratories) bring. Thus assemblies of new species may not reach the contiguity and completeness of the model genomes because of fundamental biological issues, and technical improvements and innovations, both wet laboratory and bioinformatic, are required.

Genome complexity does not appear to be closely tied to life habit. In terms of genome sizes, while some parasites (such as *Meloidogyne hapla*, with an estimated genome span of 54 Mb (Opperman *et al.* 2008)) have smaller genomes than those of free-living relatives (both cephalobes and rhabditids have genomes from 100–200 Mb), the largest genomes known thus far are in parasites (*Romanomermis culicivorax* has a genome of ~322 Mb (Schiffer *et al.* 2013), *A. suum* 334 Mb (Wang *et al.* 2012) and *Haemonchus contortus* 370 Mb (Laing *et al.* 2013)). Within clades, genome sizes can vary widely (for example within the genus *Meloidogyne* sizes range from 54 Mb in *M. hapla* to 150 Mb estimated in *Meloidogyne incognita* (Abad *et al.* 2008; Lunt *et al.* 2014)), and this is associated with polyploidy. The knowledge of genome sizes of free-living nematodes outside the Rhabditomorpha is very sparse, and it may be that free-living, diploid species’ genomes regularly exceed the two-fold range known thus far. In general, the numbers of genes predicted from parasitic species are less than or equivalent to those predicted from free-living species. *Caenorhabditis elegans* has ~21 800 protein-coding genes, while *H. contortus* (Chromadoria; Strongylomorpha) has 20 600 (Laing *et al.* 2013). However, the mermithid *R. culicivorax* (Dorylaimia; Mermithida) has ~12 000 (Schiffer *et al.* 2013), *Trichinella spiralis* (Dorylaimia; Trichinellida) has only 15 800 (Mitreva *et al.* 2011), and the onchocercid nematodes (*B. malayi* and relatives; Chromadoria; Spiruromorpha) have between 12 000 and 14 000 (Godel *et al.* 2012; Desjardins *et al.* 2013). Whether this reveals a loss of genic complexity in some parasites, or an overall more complex genomic heritage in the Rhabditina remains unclear. Changes in genome size are generally reflected in congruent changes in intron length, intergenic distance and repeat content.

## HORIZONTAL GENE TRANSFERS INTO NEMATODE GENOMES

Plant parasitic nematodes must overcome the formidable defences of the plant cell wall in order to extract nutrition from their hosts. In addition, many sedentary plant parasites induce galls of various forms, structures induced in the plant by a parasite that must ‘know’ some of the tricks of plant developmental biology. Plant parasitic species secrete cellulolytic enzymes, and cloning and sequencing of these effectors revealed that some had their closest homologues in rhizosphere fungi and bacteria rather than other animals (Smant *et al.* 1998). These enzymes became the first robustly supported instances of horizontal or horizontal gene transfer into nematode genomes. A diverse roster of plant cell wall-degrading enzymes is now known from cyst and root-knot nematodes (Bird *et al.* 2014), and supplemented by piecemeal understanding of the repertoires of other phytopathogenic species. Phylogenetic inference suggests that these horizontally transferred genes were acquired early in the evolution of the Tylenchomorpha (Danchin *et al.* 2010; Rybarczyk-Mydlowska *et al.* 2012). It is likely, given the inferred species of origin of the genes, that there were several independent events of gene acquisition (Mitreva *et al.* 2009). Surveying tylenchomorph transcriptomes and genomes for genes with sequence-similarity profiles similar to those of the validated horizontal gene transfers reveals a wide range of candidates that include *nod* factor homologues and genes of unknown function that have disjunct distributions in plants and plant-parasitic nematodes, or in root bacteria and plant parasitic nematodes (Elsworth *et al.* 2011).

Importantly, horizontally transferred plant cell wall-degrading enzymes have also been identified in parasites from the other independently evolved plant–parasitic groups (Diptherophorina in Enoplia and Dorylaimida in Dorylaimia), suggesting that while the transition to phytophagy was difficult, it involved similar evolutionary trajectories in each case: the acquisition of the necessary toolkit from professional saprophytes in the root environment. It is interesting in this respect that similar cellulolytic enzymes have been identified in the free-living *Pristionchus pacificus* and related species (Rhabditina; Diplogateromorpha) (Mayer *et al.* 2011). Horizontally acquired genes are evident in other species, including *C. elegans* (Parkinson and Blaxter, 2003).

The close association between animal parasites and their hosts, and the requirement to specifically modulate especially the adaptive and anamnestic immune responses of vertebrate hosts, raises the question as to whether animal parasites also acquired novel genes from their hosts, or other commensals of these hosts. Surveys of expressed sequence tag datasets from plant parasitic species identified many potential horizontal transfer candidates, but very few candidates were identified in either transcriptome data from across the diversity of animal parasites, or in the genomes of animal parasites in Strongylomorpha, Ascaridomorpha, Spiruromorpha or Trichinellida (Elsworth *et al.* 2011). One interesting horizontally acquired gene in the Spiruromorpha is an alphaproteobacteria-like, second copy of ferrochelatase, an enzyme involved in the synthesis of haem (Elsworth *et al.* 2011). This gene is present in onchocercid nematodes (*B. malayi* and relatives), and suggests an additional or distinct requirement for haem in these tissue and blood parasites (Wu *et al.* 2013). The ferrochelatase is quite distinct from that found in the *Wolbachia* symbionts of onchocercids (see below).

## BACTERIAL SYMBIONTS

The long history of the tree of life is punctuated by many, highly significant events of symbiosis. In the Nematoda, several distinct types of symbiosis with bacteria have been recorded (Murfin *et al.* 2012). The free-living Stilbonematidae (Chromadoria) associate specifically with gammaproteobacteria that grow as a lawn on the nematodes’ cuticle. These sulphur-fixing bacteria act as a major food source for the nematode, and the nematodes ‘farm’ their bacterial associates by migrating to H_2_S-rich sediment layers (Bulgheresi 2011; Murfin *et al.* 2012). In Anoplostoma (Enoplia), adult nematodes have neither mouth nor anus, and their guts are filled with a sulphur-fixing symbiont. Other similar trophic symbioses undoubtedly await discovery.

The entomopathogenic genera *Heterorhabditis* (Rhabditina, Strongylomorpha) (Bai *et al.* 2013) and *Steinernema* (Tylenchina, Panagrolaimomorpha) (Goodrich-Blair, 2007) share a life-cycle strategy that utilizes specific Entobacteriaciae bacterial symbionts (*Photorhabdus* with *Heterorhabditis, Xenorhabdus* with *Steinernema*) to kill newly invaded insect larvae, and then to provide nutrition to the growing and reproducing nematodes. While the symbionts used and the details of the interactions differ, the convergence of these two nematode genera on the same general strategy is remarkable. In the plant-parasitic *Xiphinema* (Dorylaimia; Dorylaimida), a veruccomicrobial symbiont, *Xiphinematobacter*, is found intracellularly (Vandekerckhove *et al.* 2000). Its role in the biology of the nematode is largely unknown, although the symbiont is maternally transmitted and may play a role in modification of the nematodes’ reproductive mode.

The alphaproteobacterium *Wolbachia pipientis* was first described from insects, where they are reproductive parasites, manipulating the reproductive status, gender or sexual compatibility of their hosts (O'Neill, 1995; Werren, 1997). *Wolbachia* have subsequently been found in a range of terrestrial arthropods, and from nematodes. Molecular phylogenetic data suggest the presence of supergroups of *Wolbachia* that have distinct biology and host distributions (Lo *et al.* 2002). Supergroup A and B *Wolbachia* are most widespread, and are found in insects. Nematodes are infected with supergroup C, D and F *Wolbachia. Wolbachia* have been described from the Spiruromorpha (in the Onchocercidae), and the Tylenchomorpha (*Radopholus similis* is the only species with infection described to date) (Jacob *et al.* 2008; Haegeman *et al.* 2009). General surveys using *Wolbachia*-specific PCR assays of many other nematode species have been negative (Bordenstein *et al.* 2003; Duron and Gavotte, 2007; Foster *et al.* 2008). However, in *R. similis* (Tylenchina, Tylenchomorpha) the identification of expressed sequence tags corresponding to likely *Wolbachia* genes led to the identification of an intracellular symbiont in this plant parasite (Jacob *et al.* 2008; Haegeman *et al.* 2009).

In supergroups A and B, the symbiont phylogeny does not match that of its hosts, and host species tend to include both infected and uninfected individuals, reflecting frequent loss and acquisition of the symbiont through phylogenetic time. This pattern reflects the parasitic nature of the symbiosis. In contrast, supergroup C and D *Wolbachia* from onchocercid nematodes show traits suggestive of long, and possibly essential, mutualist interactions. The *Wolbachia* and nematode host phylogenies are congruent, indicating little if any host switching (Bandi *et al.* 1998; Casiraghi *et al.* 2004). In infected species, all individuals are infected, and killing of the *Wolbachia* with antibiotics such as tetracyclines also affects the viability of the nematode host, with loss of fecundity and nematode death (Bandi *et al.* 1999; Hoerauf *et al.* 1999; Landmann *et al.* 2011, 2012). The exact nature of the mutualism remains unclear: the *Wolbachia* may assist the nematode metabolically (the distribution of bacteria in adult nematodes is reminiscent of the distribution of essential nutritive *Buchnera* bacteria in aphids) or in evading the vertebrate host's immune system (by confusing T-helper cell polarization with bacterial and metazoan signals at the same time) (Fenn and Blaxter, 2004, 2007; Darby *et al.* 2012). Genome sequencing of filarial *Wolbachia* has permitted culling of the possible hypotheses for essentiality, but has not yielded data that definitively support specific metabolic *vs* immunoprotective roles (Darby *et al.* 2012). It is also possible that the interference of the *Wolbachia* with oogenesis and development (Landmann *et al.* 2011, 2012) makes it difficult, in evolutionary terms, for the nematode to rid itself of the symbionts. The symbiosis is not essential in the phylogenetic long term, as there are onchocercid species, such as *Loa loa, Onchocerca flexuosa* and *Acanthocheilonema viteae*, which have lost the infection and are now *Wolbachia*-free. Genomically, filarial *Wolbachia* display the expected phenotypes of mutualist endosymbionts: the genomes are reduced compared with the insect-parasitic supergroups A and B, with fewer protein coding genes and a lack of mobile elements such as phage (Comandatore *et al.* 2013).

The onchocercid nematodes that lack living *Wolbachia* still retain a signature of past infection in the form of horizontally transferred fragments of *Wolbachia-*like DNA in their nuclear genomes (McNulty *et al.* 2013). Species that have live *Wolbachia* also have horizontally transferred *Wolbachia*-like fragments in their genomes (Dunning-Hotopp *et al.* 2007; Ioannidis *et al.* 2013). Horizontal transfer of organellar DNA to the nucleus is common, and thus the presence of these *Wolbachia* fragments could simply be a product of non-functional, stochastic incorporation of *Wolbachia* fragments into the genome (Blaxter, 2007). More excitingly, these inserted fragments could be being used by the nuclear genome to express new, *Wolbachia*-derived proteins. While some *Wolbachia* fragments are expressed at low levels (Ioannidis *et al.* 2013), most are not, and most are gene fragments that also have disabling mutations that render them inactive. Comparisons between the nuclear genomes of onchocercid species with and without *Wolbachia* has identified few shared insertions and no smoking gun of a constrained, conserved transfer that might substitute for a live *Wolbachia*.

The other *Wolbachia* found in nematodes are much less well-studied. Some onchocercid nematodes carry a *Wolbachia* that is placed in supergroup F, alongside *Wolbachia* from termites, fleas and bedbugs (Bordenstein *et al.* 2003; Duron and Gavotte, 2007; Foster *et al.* 2008; Jacob *et al.* 2008; Haegeman *et al.* 2009; Comandatore *et al.* 2013). Initial analyses suggested that the *R. similis Wolbachia* was distantly related to any other supergroup (Jacob *et al.* 2008; Haegeman *et al.* 2009), but this result is questionable (Koutsovoulos *et al.* 2014). The bovine lungworm *Dictyocaulus viviparus* (Rhabditina, Strongylomorpha) was not known to have any association with *Wolbachia* until its genome was sequenced (Koutsovoulos *et al.* 2014). Within the nuclear genome contigs were ~1 Mb of DNA fragments that had highest similarity to *Wolbachia* genomes (Koutsovoulos *et al.* 2014). These fragments bore all the hallmarks of being non-functional horizontal transfers from a once-present *Wolbachia*. Using these horizontally transferred fragments, the likely source of the transfer was identified as a supergroup F *Wolbachia*. The *D. viviparus* data allow resolution of the relationship of supergroup F (and the *Wolbachia* from *R. similis*) as sisters to supergroups C and D. The fragments in the *D. viviparus* genome included remnants of bacteriophage, suggesting that the source genome might have been more like that of the parasites of supergroup A and B than the reduced C and D symbionts. It will be important to survey other emerging nematode genomes for evidence of past association with *Wolbachia*, and perhaps other bacteria, and thus reveal the extent of the interactions between these symbionts and nematodes, and perhaps even identify particular associations with parasitism.

## A GENOME-BASED TREE OF NEMATODA

One critical issue that molecular phylogenetic analyses now face is that more data are needed. To date, most analyses have used a single locus, the nuclear small subunit ribosomal RNA gene (nSSU). The nSSU is a good gene for deep phylogenetics, but the phylogenetic history that can be extracted from its ~1800 bases is limited. There are now over 8000 nematode nSSU sequences in the public sequence databases (many fragmentary) from over 4000 nominal species. It is not possible to derive a robustly supported tree from this many sequences, and many internal nodes that were unresolved in the earliest analyses remain unresolved in the most recent ones (Holterman *et al.* 2006; van Megen *et al.* 2009; Bik *et al.* 2010), probably because of a lack of unambiguous signal in the single, short nSSU locus. The mitochondrial genome is a readily accessed source of data for phylogenetic inference, and complete genomes are available for over 40 species. These have yielded phylogenies that are well-resolved but at odds with nSSU phylogenies (Park *et al.* 2011; Sultana *et al.* 2013). Specifically, neither Spiruria (Blaxter *et al.* 1998) nor Tylenchina are recovered as monophyletic, and the sister relationship between *Heterorhabditis bacteriophora* and Strongylomorpha is not recovered (Park *et al.* 2011; Sultana *et al.* 2013). Whether these differences arise from biases or errors in the nuclear or mitochondrial data that have not been mitigated remains to be clarified.

One key utility of the new genome-wide data from a wide range of nematode species is that we have a much larger set of data to draw on when compiling matrices for phylogenetic inference (Jones *et al.* 2011). A major issue is the selection of loci that are orthologous (i.e. where representatives in different species have their origin in a single instance in an ancestral species) and where data coverage is relatively complete. Using gene sets inferred from complete genome sequences, and also complete or high-density transcriptome data, it is possible to infer a set of orthologous genes across the breadth of the phylum. One approach to achieving this is to use a tool such as Core Eukaryotic Genes Mapping Approach (CEGMA) (Parra *et al.* 2007), which identifies a set of 248 genes known to be present in six model eukaryote species. These genes tend to be highly conserved in sequence, and one limitation resulting from their use may be that there is not enough variation to record closely spaced, or recent branching patterns. An alternative approach is to generate a sequence-based clustering of all genes from all the species under study, using a tool such as orthoMCL (Li *et al.* 2003), and to query the resultant data for putative sets of orthologues. Using these approaches it is possible to build datasets that include many hundreds of thousands of aligned nucleotides (and several hundred thousand aligned codons or amino acids). These datasets can then be used to address questions left unanswered by the nSSU datasets. First attempts to explore resolution of the deeper phylogeny of Nematoda with multiple nuclear genes derived from whole-genome sequencing priojects have largely supported the existing nSSU-derived phylogeny, albeit with limited taxon representation (Desjardins *et al.* 2013; Laing *et al.* 2013).

The true power of this phylogenomic approach will only be realized when many hundred nematode genomes representing known diversity are sequenced, but already large datasets can be collated and explored. We have used a combination of whole-genome-derived and transcriptomics-derived gene sets to, firstly, attempt to replicate the findings from the nSSU analyses performed previously, and secondly to resolve some of the remaining unresolved polytomies and conflicts between analyses (Fig. 1B) (Blaxter *et al.* 2014). Analyses were performed with data from 181 genes from 23 nematode taxa including representatives of the Dorylaimia, Enoplia and Chromadoria. Taxon sampling remained most limited in Enoplia (a single representative, *Enoplus brevis*), and in the comb-like series of ordinal taxa subtending the Rhabditida in Chromadoria (only *Laxus oneistus* from Chromadorida). With these taxa the major clades (I–V) that were identified using nSSU (Blaxter *et al.* 1998) were recovered, the branching order of clades III (Spirurina), IV (Tylenchina) and V (Rhabditina) was resolved as (III, (IV,V)), and the Enoplia were robustly resolved as arising basal to Dorylaimia plus Chromadoria. While *E. brevis* has a relatively short branch length in these analyses, we caution that its placement may be artefactual due to phylogenetic artefacts elsewhere in the tree, or outgroup problems.

## PROSPECTS

The 959 Nematode Genomes initiative notes nearly 100 genomes in progress for the phylum. We have heard anecdotally of many more taxa where researchers are approaching their research goals through genome sequencing, or deep transcriptome sequencing. Improved sequencing technologies such as long single-molecule reads will improve the contiguity of genomes, and improved algorithms will enable assembly even in the presence of high levels of heterozygosity. Careful sampling, and methods for unbiased amplification of genomic DNA from single specimens will fill in the diversity of the tree, and multi-locus phylogenies provide deep resolution of relationships. The next few years will also see the development of rich collated resources for nematode genomes, including shared genome browsing environments, robust inferences of gene orthology and gene family evolution, and identification of genes and gene families that show particular patterns of evolution associated with distinct clades in the phylum. In addition, with the development of robust protocols for RNA-based interference in many species, and the development of specific genome editing methods that can be applied to any organism, we expect that questions of the specific roles of many genes to be elucidated. As ever, the questions remain biological: which traits and which genomic features are associated with parasitism, what selective forces maintain them, and how do these change through the ongoing struggle between host and parasite?
